# Estimating the Direct Cost of Cancer in Nepal: A Cross-Sectional Study in a Tertiary Cancer Hospital

**DOI:** 10.3389/fpubh.2019.00160

**Published:** 2019-06-21

**Authors:** Shiva Ram Khatiwoda, Raja Ram Dhungana, Vishnu Prasad Sapkota, Sarswoti Singh

**Affiliations:** ^1^Nepal Family Development Foundation, Kathmandu, Nepal; ^2^Department of Community Medicine and Public Health, Institute of Medicine, Tribhuvan University, Kathmandu, Nepal

**Keywords:** cancer, cost of illness, cost, health financing, Nepal

## Abstract

**Background:** The increasing prevalence of cancer and lack of strong health financing system in low income countries like Nepal is exerting an enormous financial burden on cancer patients. However, there is scant information relating to the amount of expenditure on health services for cancer treatments in Nepal. Therefore, this study aimed to estimate the direct cost associated with the treatment of cancer on the patients attending a tertiary cancer treatment center in Nepal.

**Methods:** A quantitative cross-sectional study was carried out on 294 cancer patients who were receiving treatment from Bhaktapur Cancer Hospital between 17th November 2016 and 13th February 2017. Direct medical cost and non-medical costs borne by the patients were calculated based on the cost of illness methodology. Medical cost included the cost of consultation, diagnosis and treatment while non-medical cost comprised the cost occurred out of the health facility such as the cost of food, travel, and accommodation.

**Result:** Of those 294, 169 (57.5%) were female and 125 (42.5%) were male. The median (IQR) age was 54 (19) years. Cancer of the lung was present in 19.39%, breast cancer in 15.65% and cervical cancer in 14.29%. Mean (SD) and Median (IQR) direct cost of cancer was NRs 387.5 (196.8) and 346.1 (260.5) thousand. Medical cost contributed to 80.91% of the total direct cost. Almost everyone relied on out-of-pocket (OOP) payment for cancer treatment, where 253 (86.1%) participants reported that they were experiencing financial hardship, 230 (78.2%) took a loan, and 140 (47.6%) sold their property to manage the OOP. Both medical and non-medical costs varied significantly with age, socio-economic status, types of cancer and the treatment.

**Conclusion:** Medical cost contributed the most to the direct cost. OOP was dominant payment mechanism to utilize health services. Average direct cost of cancer was higher than the average income of patients, sufficient to cause financial catastrophe. This implies the need of improved health financing strategy to protect people from the financial hazards of health service utilization for cancer in Nepal.

## Introduction

Increasing prevalence of cancer is causing a significant impact on health and finance of individuals and state, more in low income countries like Nepal (1–3). Most low income countries do not have an effective financial protection mechanism where 50% of health care financing is from OOP (out-of-pocket) payments, as compared with 30% in middle-income countries and 14% in high-income countries (4). An estimate showed that 808 million people incurred catastrophic health spending in 2010 (5), which was projected to increase with growing burden of non-communicable diseases (NCDs), including cancer, unless effective strategies are implemented (3).

The global economic burden of cancer is tremendous. It was estimated that the cost of 13.3 million new cases of cancer in 2010 was US$ 290 billion. The greatest share of expenditure was related to medical costs that accounted for US$ 154 billion (53% of the total), while non-medical costs and income losses accounted for US$ 67 billion and US$ 69 billion, respectively. The total costs were expected to rise to US$ 458 billion in the year 2030 (6). Thus, the cost of cancer is an important concern, even in developed countries (7, 8).

Like other LIMCs, health care financing in Nepal is not developed enough to protect the population from financial risk of utilizing health services in the case of chronic diseases like cancers. The partially implemented health insurance policy is abundant with several limitations and is not readily available to everyone in Nepal (9). More than 10.7% of Nepalese spend 10% of their total expenses for health, where 1.67% of the population is pushed below the poverty line of PPP$ 1.90 per capita per day (10). On the other hand, the burden of NCDs including cancer is gradually increasing in Nepal. In 2016, NCDs were estimated to account for two third of all death in Nepal, where cancer was responsible for 9% of all deaths (11). The age standardized rates in cancer incidence and mortality were estimated to be 103.7 and 77.8, respectively per 100,000 in Nepal in 2018 (12). However, due to much dependency on OOP, the existing financial hardship and impending financing catastrophe is likely to create barriers in accessing health services, undermining the importance of universal health coverage (UHC). Studies from Nepal reported that 13% of households experienced catastrophic expenditure due to OOP expenditure to health in Nepal (13) where the incidence of catastrophic health payment due to cancer was 42.9% (11.9–77.2 at 95% CI) (14). The Nepalese government is currently providing financial assistance of up to NRs 100,000 (Euro 877.19, USD 925.92) per person for cancer treatment under a scheme to support impoverished citizens (15). There is no information available whether the subsidy is sufficient to protect the patients from financial catastrophe. We found only one study which assessed cost of care of cancer patients in the same hospital in 2013. The major limitation of the study is that it restricted calculation of costs to a period of 3 months only (16).

Hence, this study aimed to calculate the direct cost of health service utilization for cancer using cost of illness (COI) methodology among cancer patients attending one of the tertiary cancer referral centers in Nepal. COI is a standard method to calculate the amount of money that was lost due to the disease and its consequences, summing the direct cost, and indirect cost (17, 18). Direct cost comprises of medical cost and non-medical cost involved for the disease under consideration. Medical cost comprises of cost of health care services, such as costs of consultation, diagnostic investigations, treatment and hospital care. Non-medical cost comprises of costs incurred out of health facility such as costs of food, travel and accommodation. This study will make a significant contribution in terms of understating the direct cost which includes medical and non-medical cost among the cancer patients in Nepal. This can also be useful to understand payment mechanisms, financial hardships of health service utilization, and different coping strategies. Findings will be useful for the families, health service providers, government and relevant agencies in regard to financial planning for management of cancer treatment (17–19) in Nepal.

## Methodology

### Methods

This was a quantitative cross-sectional study. It was conducted in Bhaktapur Cancer Hospital, one of the referral cancer centers in Kathmandu valley.

### Study Population and Sample Size

The participants were the patients attending the study site, diagnosed with any types of cancer, and had gone through investigatory and treatment processes, but not declared free of cancer at the time of study. We excluded the critically ill patients who were not able to provide the required information for the study.

Since this study is a part of another study designed to assess the health-related quality of life, the sample size calculation is originally based on estimating the true population mean of health-related quality of life in the cancer patient in one sample situation. The required sample size was 294, calculated using *n* = (z^2^ S^2^/d^2^) formula, where the standard deviation (S) and the allowable error (d) were 24.2 unit and 2.9 unit, respectively (20).

### Sampling Strategy

The study included all the eligible cancer patients who received treatment from Bhaktapur Cancer Hospital, Kathmandu between 17th November 2016 and 13th February 2017. We stopped recruiting participants once the desired sample size was achieved.

### Data Collection

A structured questionnaire was applied to collect information of the variables related to socio-demographic characteristics, diagnosis/site of cancer, duration of diagnosis, types of treatment taken, direct medical cost, direct non-medical cost, experience of financial hardship, utilization of government's support, and different coping mechanisms such as taking out loans or selling of property, etc. The tool was pretested in 30 patients in a similar setting and finalized based on the feedback from the pretest before applying it in the study. During a face to face interview, the participants and their caretakers reported the direct medical and non-medical costs they had to spend from the beginning of service utilization up to the time of interview. Information provided by the participants on the cost was cross-checked with their relevant documents and the hospital's records.

Using the cost of illness (COI) method, respective component costs were added to find medical cost and non-medical cost, and finally both of them were added to calculate the resultant direct cost (17, 18). Direct cost included the cost involved directly due to the disease. It comprised of medical and non-medical costs. Medical cost comprised of costs of consultation, diagnosis and investigations and hospital care, and treatment taken, such as chemotherapy, radiotherapy, surgery, or palliative and supportive care. Non-medical cost on the other hand comprised of costs of food, travel and accommodation during health service utilization for cancer. We only aimed to calculate direct cost (medical and non-medical costs) due to cancers, but not the indirect cost (such as productivity lost etc.). As the participants were under treatment of cancer at the time of the study, the study could only assess on the past and present expenditure. The study was not designed to follow up the participants; therefore, it missed the information on expenses beyond the date of study enrollment of the participant.

### Data Analysis

Data were entered in epi-data 3.1, and analyzed in IBM SPSS 21. Descriptive statistics such as percentage, mean, standard deviation (SD), median and interquartile range (IQR) are presented in tables. We used non-parametric tests, Mann-Whitney *U*-Test and Kruskal-Wallis Tests, to test the group variation of the cost.

### Ethical Consideration

This study obtained ethical approval from the Institutional Review Board (IRB) of the Institute of Medicine (IOM). We also sought for written permission from Bhaktapur Cancer Hospital. Participants were well-informed on the study objective and procedures and were assured that we would safeguard their privacy and ensure confidentiality of the information they provided. Written informed consent was also obtained from the participants before the study commencement.

## Results

Total 294 participants responded to the interview questionnaires. Most of the participants belonged to age group 50–59 years (*n* = 99; 33.7%). Median (IQR) age of the participants was 54 (19) years. There were 169 (57.5%) female and 125 (42.5%) male participants (Table 1).

**Table 1:** Socio-demographic characteristics of participants (*n* = 294).

**Characteristics**		***n***	**%**
Age group	≤29 years	14	4.8
	30–49	90	30.6
	50–59	99	33.7
	60+	91	31.0
Sex	Female	169	57.5
	Male	125	42.5
Residence	Rural	182	61.9
	Urban	112	38.1
Marital status	Married	253	86.1
	Unmarried/others	41	13.9
Religion	Hindu	249	84.7
	Buddhist and others	45	15.3
Ethnicity	*Janajati/Dalit*/others	177	60.2
	*Brahmin/Chhetri*	117	39.8
Family type	Nuclear	146	49.7
	Joint or extended	148	50.3
Education	Primary or below^§^	187	63.6
	Above primary	107	36.4
Occupation	Informal^¥^	141	48.0
	Formal^†^	62	21.1
	Student/Unemployed	91	31.0
Monthly income	Up to 20 thousand	134	45.6
	Above 20 thousand	87	29.6
	Not mentioned	73	24.8
	(Mean NRs 18,278.91, median 15,000.00)		
Economic status (self-reported)	Enough to for a year	166	56.5
	Not enough for a year	99	33.7
	Extra saving	29	9.9

§ *Formal schooling up class five was considered primary education in this study*.

¥ *Informal occupation included household works, small scale agriculture and livestock farming, small shop keeping, labor, etc*.

† *Formal occupation included registered/official employment or formal business etc*.

Table 2 shows the types of cancer, payment mechanism and experience of financial hardships for health service utilization. Regarding diagnosis (cancer site) of the cancer, 57 (19.93%) were identified with lung cancer, 46 (15.65%) had breast cancer, and 42 (14.29%) suffered from cervical cancer, and 149 (50.68%) had other cancers.

**Table 2:** Characteristics related to disease, payment mechanism and experience of financial hardship of the participants (*n* = 294).

**Characteristics**		***n***	**%**
Diagnosis (cancer site)	Lung	57	19.39
	Breast	46	15.65
	Cervical	42	14.29
	Others^ψ^	149	50.68
Stage of cancer	I	14	4.8
	II	21	7.1
	III	17	5.8
	IV	13	4.4
	Not mentioned	229	77.9
Duration of diagnosis	<6 months	123	41.8
	6 months and above	171	58.2
	Median (IQR) duration of diagnosis: 6.5 (7) months		
No. of facility visited	Up to three	170	57.8
	Four or more	124	42.2
	Median (IQR) number of facility visited: 3(3)		
Present treatment	Chemotherapy	163	55.4
	Radiation therapy	28	9.5
	Surgery	12	4.1
	Palliative therapy	6	2.0
	Others*^K^*	85	28.9
Government's support^ϕ^	Utilized	97	33.0
	Not utilized	197	67.0
Method of payment	Person/Household (Out-of-pocket)	191	65.0
	Both household and government	97	33.0
	Others (charity, insurance, etc.)	6	2.0
Loan taken	Yes	230	78.2
	No	64	21.8
Sold property	Yes	140	47.6
	No	154	52.4
Experiencing hardship	Yes	253	86.1
	No	41	13.9

ψ *Other cancers: Gastrointestinal (related to stomach, intestine, GB, Liver) 42 (14.3%), Oral/Neck/Brain related 37 (12.6%), Blood and lymph related 19 (6.5%), and others (bone, muscle, penile, prostate, UB, ovary etc.) 51 (17.3%)*.

ϕ *Asked if the participants had utilized the government's subsidy for medical care at the time of interview*.

K *Includes treatment other than chemotherapy, radiotherapy, surgery and palliative*.

Most of the patients (*n* = 229, 77.9%) did not have the record of the stage of cancer. Regarding present treatment, 163 (55.5%) were receiving chemotherapy, 28 (9.5%) were receiving radiation therapy, 12 (4.1%) had undergone surgery, 6 (2%) were on palliative care, 85 (28.9%) other treatments at the time of study in the hospital.

Regarding payments of cancer treatment, all participants incurred the cost in out-of-pocket (OOP) expenses, where 191 (65%) of the participants expended solely themselves, 97 (33%) received a government subsidy and 6 (2%) used other mechanisms to partially cover the health care cost. Most of the participants (86.1%) experienced financial difficulty during the course of treatment. To cope with financial hardship 230 (78.2%) participants took out a loan to assist in health service utilization and 140 (47.6%) of them sold their property.

**Figure 1:**
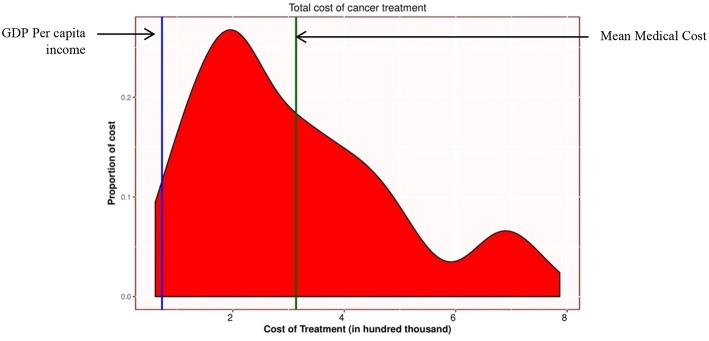
Distribution of medical cost (in NRs ‘000). Green vertical line represents the mean medical cost NRs 313,000.00. (Euro 2,745.6, USD 2,898.14). Blue vertical line represents the per capita income NRs 78,946.00 (Euro 692.5, USD 730.98) (22). During study period (as of 1st Jan 2017), exchange rate for one Euro was NRs one hundred fourteen, and for one US$ was NRs one hundred and eight (21).

Table 3 shows the direct cost of the participants utilizing health care services. Mean (SD) and median (IRQ) direct cost of cancer was NRs 387.5 (196.8) and 346.1 (260.5) thousand. Medical cost contributed to 80.91% of the total direct cost. Mean (SD) and median (IQR) medical cost of cancer was NRs 313.54 (178.29) and 263.65 (238.1) thousand (Figure 1). Treatment contributed 72.40 % of the total medical cost. The mean (SD) and median of cost of treatment was NRs 227.01 (140.48) and 310.95 thousand. Likewise, the mean (SD) and median cost of diagnosis and investigations was 45.20 (23.68) and 52.50 thousand. And the mean (SD) and median cost of consultation (including the cost of hospital care) was 41.37 (47.03) and 47.50 thousand. The mean and median (IQR) non-medical cost of cancer was NRs 73.99 (SD 39.39), and 66.00 (40.6) thousand. The mean (SD) and median cost of food and accommodation was 49.99 (39.66) and 66.00 thousand. And the mean (SD) and median cost of travel was 24.58 (22.67) and 16.95 thousand. Minimum direct cost of the participants receiving treatment for cancer was 91.5 thousand and the maximum was 996.2 thousand.

**Table 3:** Total direct cost (NRs, ‘000) of cancer (*n* = 294).

**Direct cost of cancer (% of total direct cost)**	**Mean**	**SD**	**25th percentile**	**Median**	**75th percentile**	**Minimum**	**Maximum**
Medical costs (80.91 %)	313.54	178.29	181.20	263.65	419.40	60.91	786.7
Cost of treatment including medicine, therapies and surgeries	227.01	140.48	112.25	197.87	310.95	24.0	611.0
Cost of diagnostic investigations	45.20	23.68	28.87	40.05	52.50	8.5	138.1
Cost of consultation and including hospital care	41.37	47.03	12.13	20.06	47.50	1.8	184.6
Non-medical cost (19.09 %)	73.99	39.66	49.10	66.00	63.90	11.7	251.5
Cost of food and accommodation	49.40	28.02	28.95	45.60	63.77	6.0	173.7
Cost of travel	24.58	22.67	7.50	16.95	34.50	1.4	130.0
Total direct cost (sum of medical and non-medical costs) (100 %)	387.54	196.81	243.31	346.10	503.81	91.5	996.2

*During study period, exchange rate of one Euro was NRs one hundred fourteen, and of one US$ was NRs one hundred and eight (21)*.

Table 4 shows the bivariate analysis. Direct cost was statistically significant with age group, economic status on the basis of sufficiency to feed the family (as reported by the participants), diagnosis (site of cancer), duration of diagnosis, type of treatment. Direct cost of the age group 30–49 years was higher, followed by age group 50–59 years, age group ≤ 29 years, and 60 years or above. Among the major three cancers, mean direct cost of cervical cancer was highest, followed by breast and lung [NRs 410.67 (SD 180.58), 407.02 (SD 153.21), 247.87 (SD 131.36) thousands, respectively]. Similarly, those whose treatment duration was of 6 months or above had higher mean direct cost [NRS 426.22 (SD 194.72) thousand] than those who had duration <6 months [NRs 333.75 (SD 187.56) thousand]. On the basis of types of treatment at the time of interview, the participants who were receiving surgical treatment had lesser mean direct costs than those receiving chemotherapy, radiotherapy and palliative care [NRs 327.00 (SD 50.08), 346.32 (SD 191.09), 347.52 (SD 170.69), 416.19 (SD 132.16) thousands, respectively]. Those who used government's support of one lakh had higher medical cost (NRs 331.42 thousand) than those who had not utilized it (NRs 304.73 thousand), at the time of interview.

**Table 4:** Bivariate analysis of direct cost (NRs ‘000) of cancer (*n* = 294).

**Characteristics**		**Total medical cost**		***P*-value**	**Total non-medical cost**		***p*-value**	**Total direct cost**		***p*-value**
		**Mean (SD)**	**Median**		**Mean (SD)**	**Median**		**Mean (SD)**	**Median**	
	≤ 29 years	323.34 (183.34)	312.98	<0.001^*^	54.15 (35.18)	41.40	<0.001^*^	377.50 (199.28)	353.22	<0.001^*^
Age	30–49	354.94 (181.43)	328.41		85.62 (47.30)	77.10		440.56 (203.70)	383.83	
	50–59	329.61 (190.20)	275.50		78.09 (37.02)	70.70		407.71 (209.02)	357.76	
	60+	253.61 (145.07)	208.95		61.08 (27.91)	57.20		314.69 (152.06)	271.20	
Economic status (self-reported)	Not enough	268.03 (187.45)	196.31		63.72 (35.35)	56.20		331.75 (202.08)	259.06	0.001^*^
	Enough	352.03 (173.87)	318.33	<0.001^*^	76.99 (40.73)	67.40	0.001^*^	429.02 (195.72)	381.98	
	Extra saving	248.58 (105.07)	215.90		91.93 (36.42)	93.00		340.51 (114.74)	297.70	
Diagnosis	Lungs	195.55 (128.90)	169.06	<0.001^*^	52.31 (25.06)	46.90	<0.001^*^	247.87 (131.36)	228.06	<0.001^*^
	Breast	321.94 (136.21)	263.65		85.08 (46.91)	73.75		407.02 (153.21)	381.05	
	Cervix	333.44 (167.62)	298.06		77.22 (38.84)	66.87		410.67 (180.58)	363.16	
	Others	350.47 (190.66)	318.11		77.95 (38.84)	69.10		428.43 (210.99)	385.75	
Duration	<6 months	269.48 (174.16)	211.60	<0.001^*^	64.26 (31.39)	57.80	0.001^*^	333.75 (187.56)	289.45	<0.001^*^
	6 months and above	345.23 (174.94)	326.81		80.99 (43.00)	69.10		426.22 (194.72)	395.01	
Present treatment	Surgery	249.80 (54.12)	242.80	<0.001^*^	77.20 (23.61)	84.05	<0.001^*^	327.00 (50.08)	324.70	<0.001^*^
	Radiotherapy	287.54 (156.33)	210.40		59.98 (23.59)	57.20		347.52 (170.69)	249.41	
	Chemotherapy	279.48 (176.82)	220.10		66.83 (37.38)	59.20		346.32 (191.09)	289.45	
	Palliative care	338.26 (119.90)	378.78		77.93 (17.21)	88.70		416.19 (132.16)	453.33	
	Others	394.66 (178.13)	360.29		91.61 (44.34)	76.40		486.28 (199.19)	423.76	
Government's support	Utilized	331.42 (169.22)	310.56	0.124	75.03 (40.91)	64.10	0.828	406.46 (187.03)	385.50	0.132
	Not utilized	304.73 (182.37)	242.80		73.48 (38.71)	67.30		378.22 (201.26)	326.50	

* *p <0.05*.

## Discussion

This study assessed the direct cost involved during healthcare service utilization for cancer in a tertiary care hospital in Nepal. Our study found that average direct cost of the cancer was higher than average income of Nepalese people, sufficient to cause financial catastrophe. Most of the direct cost was due to medical cost. Most of the cost was borne by household though out-of-pocket (OOP) payment mechanism. Many cancer patients experienced financial difficulty and had to take loan or sell property. This situation is similar to many developing countries. Total direct cost, medical cost and non-medical cost varied significantly with age, socio-economic status, types of cancer and the treatment. The finding implies the need for a better strategy to protect people from financial hardship due to cancer.

The study finding suggests that the average direct cost of cancer care (NRs 387,000) and average medical cost (NRs 313,000) were far above the average annual income of a person (NRs 78,946.00) (22) (Figure 1). According to WHO, financial catastrophe occurs when healthcare payment is at 40% or more of a household's capacity to pay (non-food expenditure) in a year (23, 24). Some scholars also assume that healthcare cost that exceeds 10% of annual household income causes financial catastrophe (25). According to Fifth Household Budget Survey 2014/15, 40% of capacity to pay (non-food expenditure) and 10% of annual income of Nepalese household were NRs. 69,398.40 and NRs. 36,145.2, respectively (26). The average direct cost of cancer was higher than the capacity to pay of the Nepalese household. Therefore, that direct cost of cancer could cause financial catastrophe to the families of cancer patients.

Most of them 253 (86.1%) reported that they experienced financial difficulty due to cancer treatment. To cope with the financial burden, majority of participants took a loan (78.2%), and some even sold their property (47.6%). According to a study done by ACTION Study Group, cancer diagnosis in Southeast Asia is disastrous, with over 75% of patients experiencing death or financial catastrophe within 1 year (27). In a study conducted in Pakistan, the financial burden of cancer was mostly borne by the patient or the family, 42% of patients perceived the burden as significant and 27% patients perceived it as unmanageable. Most of the time, the average monthly cost of treatment far exceeded the monthly household income (28). In Vietnam 37.4% of the households with cancer sufferers were impoverished by the treatment costs (29). Despite having free medical care for breast cancer, a study in Haiti found that two-thirds of women suffered financial catastrophe because of the OOP expenditure for non-medical cost and medical cost for out of facility care (30), and 52% of the participant suffered debt and 20% sold possessions (31).

Compared to our finding (median direct cost = NRs 346.1 thousand), a study conducted in the same setting in Nepal in 2013 reported lesser direct cost incurred in cancer treatment (median cost = NRs 149.7 thousand) (16). This could be due to an increase in the cost of cancer treatment or because of lesser duration of service utilization in the previous study. Rising cost of cancer medicine is a concern for many countries (7). Cancer treatment is considered as the most expensive healthcare service in neighboring country India (32). The cost of cancer care is increasing in China (33). Financial hardship due to cancer is also an important agenda in developed countries like USA, where 42.4% people used up their entire life's assets 2 years after the diagnosis of cancer (8, 34). Many households face catastrophic health expenditure and impoverishment as a result of the spending for chronic disease including cancer (35, 36).

In this study, almost everyone relied on household's out-of-pocket (OOP) payment at the time of service utilization for cancer. People need to put money forward from their pocket to initiate utilization of health service in Nepal. OOP expenditure in health puts families into economic hardship, threatens household's financial capacity to maintain subsistence needs, and prevents their overall wellbeing for the long term (37). Lack of prepayment or health insurance, availability of health services requiring payment, and low capacity to pay are preconditions to catastrophic health expenditure (24). Growing NCDs, dominant OOP and lack of financial risk protection and subsequent financial catastrophe in many low and middle income countries are implying the need of better health financing (13, 23, 25, 35, 36).

In Nepal primary health care is free in government health facilities. But in private facilities and for diseases like cancers people have to pay through OOP. The recently launched health insurance program may lay the foundation for sustainable health financing (15, 38) and may yield a reduction in financial hardships by reducing much reliance on OOP. For this, much insight can be obtained by analyzing the context and experience of previous community based health insurance programs (9, 39). There is a government's subsidy to medical care which provides NRs 100,000 (USD 925.92, Euro 877.19, exchange rate as of 1st January 2017) through a scheme of support to impoverished citizen for cancer treatment (15). The subsidy is limited, and as findings from our study suggest, insufficient to protect people, especially those from lower socioeconomic households, from financial catastrophe due to health service utilization for cancer. At the time of study only 97 (33%) participants had utilized government's subsidy. One of the reasons of few people utilizing the subsidy at the time of study could be due to the time taken by complex process of receiving a diagnosis in a health facility, and taking recommendation from local as well as district administration. Based on those recommendations, hospital will finally provide medical services up to the limit of subsidy. Hospitals get the reimbursement from the health department of health ministry. Some participants might have been considering initiating the process of utilizing the subsidy. Lack of knowledge about the subsidy may also be another barrier in utilizing it.

Addressing hardships caused by cost of cancer and other barriers of health service utilization is a concern in many countries (7, 8, 40). Different strategies can be adopted to reduce financial hardships of the patients. Possible opportunities to improve health financing may include expanding governmental financial support and strengthening prepayment mechanisms like health insurance programs (4, 23, 36, 41). Increasing tax on harmful substances like tobacco can provide funds for cancer care as well as helping in cancer prevention (42). Avoiding low value therapies may also help in protecting people and government from financial burden (43). According to this study most of the direct cost was due to medical cost (80.91%) which was mostly due to treatment cost (72.40%). Total direct cost, medical cost, and non-medical costs were statistically significant with age, socio-economic status, types of cancer and the treatment. This information can be useful to adjust the allocation of resources for cancer care. The outcome of such strategies can lead to assurance of well-being of people by ensuring universal health coverage (UHC) and preventing poverty, both of which are sustainable development goals (SDGs) (44).

Besides improving financing to healthcare, we need to implement better preventive programs and strengthen curative services to improve the health of people (45, 46). Subsequently, this may reduce the cost of healthcare by preventing disease complication and increasing productivity. Different activities for prevention including early diagnosis and treatment, such as mass awareness campaigns, HPV vaccination, enabling women to breast self-exam, improving hygiene and sanitation, motivational support to quit tobacco and smoking, etc. should also be the focus of policy and programs (47–49).

There are some limitations in the study. Since the participants were under treatment, the overall cost of complete treatment could possibly be higher than as reported in the study. There could be recall bias when sharing information about the cost of cancer from the participants. The study was done only in one hospital, which is managed by non-profit organization in collaboration with government. The cost of care of the people utilizing treatment in private hospitals or abroad can even be higher. Since we lack a proper database of financial activities of cancer patients, longitudinal study can be more helpful to assess financial concerns of people who have been utilizing health services for cancer.

## Conclusion

This study calculated direct cost of the people utilizing health service for cancer. Most share of the direct cost was due to medical care. The cost can be even higher if service utilized in private hospital or abroad. Average direct cost of cancer was higher than the average income (capacity to pay) of the people and payment for health service utilization was dominated by the OOP mechanism. Direct cost of cancer alone was sufficient to cause financial catastrophe. Existing government subsidies alone cannot cover the cost of cancer care to people in much need, especially those with a low socioeconomic status. Total direct cost, medical cost, and non-medical costs were statistically significant with age, socio-economic status, types of cancer and the treatment. This information can be useful to adjust during the allocation of resources for cancer care. To protect people from cancer and subsequent financial hardships, improved health financing along with better preventive and curative strategies have to be adopted. Possible opportunities to improve health financing may include expanding government's financial support, strengthening health insurance program, and increasing tax on harmful substances like tobacco to fund cancer care.

## Data Availability

Data used to support the findings of this study are available from the corresponding author upon request.

## Ethics Statement

This study obtained ethical approval from the Institutional Review Board (IRB) of the Institute of Medicine (IOM), Tribhuvan University, Kathmandu, Nepal. We also sought for written permission from Bhaktapur Cancer Hospital and written informed consent from the participants before study commencement.

## Author Contributions

SK conceptualized, executed the study, interpreted the findings, and prepared the first draft. RD analyzed and interpreted the findings, and drafted the manuscript. VS and SS conceptualized the study and interpreted the findings. All authors read and approved the final manuscript.

### Conflict of Interest Statement

The authors declare that the research was conducted in the absence of any commercial or financial relationships that could be construed as a potential conflict of interest.

## Abbreviations

COI: Cost of Illness
IQR: Inter quartile range
LMICs: Low- and Middle-Income Countries
NCDs: Non-communicable Diseases
NRs: Nepalese Rupee (Nepalese Currency)
OOP: Out-of-pocket
PPP: Purchasing Power Parity
SD: Standard Deviation
SDGs: Sustainable Development Goals
UHC: Universal Health Coverage
WHO: World Health Organization.
